# Improving hand sensibility in vibration induced neuropathy: A case-series

**DOI:** 10.1186/1745-6673-6-13

**Published:** 2011-04-27

**Authors:** Birgitta Rosén, Anders Björkman, Göran Lundborg

**Affiliations:** 1 Department of Hand Surgery, Skåne University Hospital Malmö, Sweden

## Abstract

**Objectives:**

We report a long-term series of nine workers suffering from vibration-induced neuropathy, after many years of exposure to hand-held vibrating tools at high or low frequency. They were treated with temporary selective cutaneous anaesthesia (EMLA^® ^cream) of the forearm repeatedly for a period up to one year (in two cases four years). The aim was to improve their capacity to perceive touch and thereby improve hand function and diminish disability. The treatment principle is based on current concepts of brain plasticity, where a deafferentation of a skin area results in improved sensory function in adjacent skin areas.

**Methods:**

All participants had sensory hand problems in terms of numbness (median touch thresholds > 70 mg) and impaired hand function influencing ADL (mean DASH score 22).

After an initial identical self-administered treatment period of 8 weeks (12-15 treatments with increasing intervals) they did one treatment every 2-3 month.

**Results:**

After one year sensibility (touch thresholds and tactile discrimination) as well as hand function (mean DASH score 13) were improved in a majority of the cases. Seven of the participants choose to continue the treatment after the first year and two of them have continued at a regular basis for up to four years. A surprising, secondary finding was diminishing nocturnal numbness of the hand and arm in eight of the nine subjects from "frequently" to "hardly ever or never".

**Conclusions:**

Our observations open new perspectives for treatment of impaired sensibility and hand function in a group of patients with vibration induced hand problems where we have no treatment to offer today.

## Introduction

The use of handheld vibrating tools is common in many occupations where low- as well as high frequency vibrating tools is used. However, long-term use of vibrating tools constitutes an occupational health risk, and various neurological and vasospastic symptoms are common, for example sensory disturbances, pain, intolerance to cold, reduced grip strength, and reduced tactile dexterity with fumbling [1-4]. Hand function may be severely impaired over the years and may affect the ability to work and quality of life [5-7]. In the long term perspective vibration exposure can lead to hand-arm-vibration syndrome, a complex condition with sensory and musculoskeletal as well as vascular symptoms [4]. Vibration-induced neuropathy is sometimes combined with carpal tunnel syndrome, and it is often difficult to differentiate between them [8,9]. Apart from ergonomic measures there is, at present, no effective treatment for the neuropathy.

Peripheral nerve injury and neuropathy result in changes in the central nervous system [10,11], and we have demonstrated, using magnetic resonance technique (fMRI), that also patients exposed to handheld vibrating tools and with sensory disturbances in their hands also show cortical changes with significantly larger activated cortical volumes and fusion of digital representations in the primary somatosensory cortex (S1) [12,13]. The findings are similar to those in conditions such as hand dystonia [14,15] with loss of motor control in individual fingers following long time repetitive synchronous movements of the digits [15]. Such functional impairments are probably the result of cortical reorganisation following long-term non-physiological sensory input [16] and they may also partly explain the symptoms seen in patients with vibration-induced neuropathy.

All parts of the body are represented in a sensory cortical body map [17] which is constantly modulated in response to the afferent nerve impulses [18-20]. Increased tactile experience or acute deafferentation of a body part results in rapid cortical reorganisation [20,21].

Treatment with temporary selective cutaneous anaesthesia using an anaesthetic cream (EMLA^®^) containing 2.5% lidocain and 2.5% priolocain, AstraZeneca, Södertälje, Sweden) on the forearm to improve the sensory function of the hand, is a new method based on rapid cortical plasticity [22]. The EMLA^® ^treatment concept is an example of guided plasticity [23] where the ability of the CNS to change is used for therapeutic purposes. Cutaneous anaesthesia of the forearm results in expansion of the cortical hand area in the S1 and thus more nerve cells being made available to the hand. In healthy subjects we have used fMRI technique to demonstrate that cutaneous anaesthesia of the forearm skin induces a cortical reorganisation, resulting in expansion of the cortical hand representation. In conjunction with the cortical expansion the sensory function of the hand improved significantly [24].

Application of EMLA^®^) to the forearm is a simple and non-invasive method that results in a deafferentation of the forearm in the somatosensory cortex which allows the hand to expand on the forearm area in the S1, resulting in more nerve cells supplying the hand [24]. Clinically the sensory function of the hand improves following EMLA^®^) application to the forearm [22,24,25]. The method works in the upper as well as lower extremity in healthy individuals and also enhances the effect of sensory re-education in patients following peripheral nerve repair [22,25,26]. Earlier we described application of the method in a single-case of vibration-induced neuropathy [27]. However, the long-term effect is of interest, and here we present a series of nine pilot cases in which the method has been used for up to four years.

## Methods

### Selection of Cases

During 2006-2009 a total of fifteen consecutive patients with a history of neuropathy induced by long term exposure to vibrating handheld tools were referred to the Department of Hand Surgery in Malmö with the specific question: "is EMLA^® ^treatment a possible way of improving the impaired hand function?" (Table 1).

**Table 1:** Demographic and clinical characteristics at baseline

Subj nr	Gender	Age	Years of exposure	Nocturnal numbness	Touch threshold at fingertip level* (g, median)	Tactile discrimination at fingertip level (2PD,median)**	Dexterity (Purdue pegboard) ***	Disability (DASH-score) ****
1	Female	65	19	frequent	0.07	4	16	9

2	Female	65	25	sometimes	0.4	≥16	5	33

3	Female	48	29	sometimes	0.16	3.7	16	7.5

4	Female	47	20	frequent	0.16	3.4	13	39

5	Male	64	45	frequent	0.4	4	8	37

6	Male	57	40	hardly ever/never	0.16	4.3	13	26

7	Male	30	13	frequent	0.16	3.4	18	14

8	Male	37	15	sometimes	0.04	3.4	14	14

9	Male	60	40	sometimes	1.0	10	10	11

* Semmes-Weinstein monofilaments (20 probes 0.008 g - 300 g), normal ≤ 0.07 g

** Two-point discrimination, normal ≤ 5 mm

*** Number of pegs picked up and placed during 30 seconds

**** Disability of the arm, shoulder and hand, normal ≤ 10

They patients had impaired touch thresholds in the fingers as measured with Semmes-Weinstein monofilaments (> monofilament # 2.83 = 70 mg), and subjective sensory disturbances with e.g. dyscoordination and impaired dexterity.

EMLA^® ^treatment specifically addresses sensory disturbances, and five of the referred cases were excluded at an early stage and subjected to further examination or other treatment, because their problems were not primarily sensory.

After the initial treatment period of eight weeks, nine of the patients showed improved touch thresholds and tactile discrimination, they also subjectively experienced improvement and wanted to continue the treatment. In one patient sensory function did not improve at all - subjectively or objectively - and there was no wish to continue the treatment.

In the group of nine patients who wanted to continue EMLA^® ^treatment, four had been exposed to high-frequency vibrating tools (dental hygienists, dental technicians), and five had been exposed to low-frequency vibrating tools (car or lorry mechanics, road construction worker). Two of the participants had retired and seven were in full-time employment, one worked part-time for six months in the middle of the treatment period (subject no 7). Demographics at referral are shown in Table 1.

The study was conducted according to the Helsinki Declaration, the local ethics committee approved the study design, and all participants gave their consent.

### EMLA^® ^treatment and follow-up

We used a recently presented protocol for treating hand conditions with diminished sensory function (minimum protective sensibility) using a treatment programme in which the skin of the forearm is repeatedly anaesthetised by cutaneous application of EMLA^® ^cream [25]. Prior to the treatment a medical history was taken focusing on adverse effects from prior use of local anaesthetic agents, including allergic reactions. Vibration-induced neuropathy is frequently bilateral and EMLA^® ^was applied to the arm subjectively considered to be the most symptomatic. The cream was applied under an occlusive bandage, for 90 minutes to the flexor aspect of the forearm, over an area stretching from the wrist and 15 cm proximally. After 90 minutes, the EMLA^® ^was carefully washed off.

This initial treatment showed improvement in sensory functions with improved touch thresholds and/or improved tactile gnosis (Table 2). The nine persons were then offered an 8-week treatment period (12-15 EMLA^® ^applications in descending frequency) based on self-administered treatments and regular follow-ups. This entailed three treatments in the first week, two treatments in weeks 2-6, and one treatment in weeks 7-8. Subsequently, "booster doses" were given once every month over one year if the patient experienced improvement.

**Table 2:** Result after treatment over 1 year with EMLA^® ^cream in nine persons with subjective problems after long-term exposure to hand-held vibrating tools

	Pre treatment	After 1 treatment	After 8 weeks (15 treatments)	After 1 year (one treatment every 2-3 months)
**Touch thresholds **(Semmes Weinstein monofilaments, g) Normal ≤ 0.07 g median	0.2 (0.04-0.5)	0.04 (0.04-0.5)	0.04 (0.04-0.2)	0.04 (0.02-0.07)

**Tactile discrimination **(2 point discrimination, mm), normal ≤ 5 mm mean	5.8 (3.4 - ≥16)	3.3 (2.2-5.0)	3.8 (2.8-8.0)	3.1 (2.2-5.0)

**Dexterity **(Purdue Peg Board, number of pegs picked and placed), mean	13 (5-18)	Not evaluated	13 (9-18)	15 (10-17)

**Disability **(DASH-score, normal below 10), mean	22 (8-39)	Not evaluated	Not evaluated	13 (0-32)

**Grip strength **(Jamar, second position, kg), mean	37 (16-62)	Not evaluated	38 (22-62)	41 (28-67)

**Nocturnal numbness **(Number of subjects)				
				
Frequently:	4	Not evaluated	0	0
Sometimes	4		2	1
Hardly ever/never	1		7	8

Median or mean are registered, and range given within brackets.

The participants were also instructed to perform a simple sensory re-education program [28] several times a day during the initial eight-week treatment period.

Assessment of hand function was performed according to standardised methods (ASHT, 1992), at regular intervals for up to one year after initiating the treatment.

A full set of 20 Semmes-Weinstein Monofilaments (SWM) was used for assessment of touch thresholds at fingertip level, two-point discrimination (2PD) at the tips of digits II and V for assessment of tactile discrimination (carried out according to the "Moberg method" [29]) in a descending order, starting with 15 mm to assess the level at which responses were correct. Dexterity was assessed using Purdue Pegboard [29], and grip strength using Jamar dynamometer [29]. For assessment of disability the DASH (disability of the arm hand and shoulder) questionnaire was used [30]Assessments were performed prior to and after initial treatment, after 8 weeks, 6 months and 1 year.

### Analysis

The majority of the data was ordinal and a nonparametric statistical method - Wilcoxon signed rank test - was used to calculate the changes in status before treatment and after one year. Due to the small sample size only two time points - before treatment and after one year - were calculated.

## Result

The results for the nine subjects after treatment for one year are summarised in Table 2. Touch thresholds improved significantly (p = 0.01), as did level of disability expressed in a lowered DASH-score (p = 0.03). A 10-point decrease in the DASH score is considered a clinically meaningful change [31], and the mean change in the present study was 9.

Dexterity improved in five of the participants, however not significantly on group level. No changes were seen in grip strength.

After one year eight of the nine subjects decided to continue the treatment with applications of EMLA^® ^every 2-3 months.

Subject # 1 has previously been described in a case report and like subject # 5, has now undergone the treatment with EMLA^® ^on a regular self-administered basis for four years. They, as well a majority of the group, said that they can "feel" when a treatment is needed, depending on level of manual activity and season. Sensory status is normally better during warmer periods.

## Discussion

This case series shows that repeated cutaneous application of an anaesthetic cream, EMLA^®^, to the volar aspect of the forearm for 90 minutes in 15 treatments during an eight-week period, results in subjectively, as well as objectively, improved hand function and reduced disability in persons with vibration-induced neuropathy. With booster doses the result lasted at least one year. After one year eight of the nine subjects to date decided to continue the treatment with applications of EMLA^® ^every 2-3 months, and two participants have continued treatment for up to four years, maintaining the result.

Extensive simultaneous sensory or motor stimulation of the digits can produce a use-dependent cortical reorganisation of digital receptive fields. There are several situations where an unfysiological sensory input eventually induces a reorganisation of the cortical hand map in analogy with that which can be found after long term work with hand-held vibrating tools [14,32,33]. In such situations the cortical hand map is distorted and rearranged into a disorganized pattern.

In humans, normal stimulation on the fingers generated by everyday use of the hands is non-simultaneous. Studies on primates [16,34] and humans [35] have shown that synchronous stimulation of multiple digits results in a breakdown of the normally sharply segregated representations of adjacent digits in the primary somatosensory cortex to multiple-digit receptive fields covering two or three adjacent digits.

We have previously shown, using fMRI at 3T, that the activated volumes in the hand area of the primary somatosensory cortex were significantly larger in persons with neuropathy due to long-term exposure to high-frequency vibrations than in healthy controls. The normal finger somatotopy was changed to a pattern where the individual fingers overlap each other. Furthermore, activation in the primary motorcortex was weaker and more scattered in the neuropathy group compared to controls [13].

Cutaneous local application of an anaesthetic cream, where the deafferented area can be precisely controlled, is a simple treatment. Following an initial application session at the hospital, self-administration is possible at home for a well-informed patient. Care has to be taken as the protective sensation on the forearm is impaired for a few hours after the application of the cream. The anaesthetic agent EMLA^® ^has been widely tested, and has no, or few, known side effects [36]. In the present group one of the subjects had a rash on the skin for a few hours after one of the applications. In routine practice, temporary anaesthesia using EMLA^® ^is followed by rapid and complete normalisation of all sensory functions in the body-part treated [36].

The clinical effect of the deafferentation is already obvious 90 minutes after application indicating that unmasking of pre-existing synaptic connections is the mechanism underlying the rapid modulation of sensory function [37].

A surprising, but interesting, finding was that the treatment acted positively on carpal tunnel-like symptoms such as nocturnal numbness (Table 2), which is a common problem in vibration-induced neuropathy [8]. Although difficult to explain this is probably also an effect of the cortical reorganisation induced by the cutaneous anaesthesia.

The well-developed feedback system between the hand and the brain, with continuous proprioception and tactile input that are coordinated with memory systems in the brain, is a prerequisite for regulation of grip force and grip speed [38] Restoration of this feedback system is one of the aims after EMLA^® ^treatment and possibly explains the improved functional use of the hand. The training of discriminative sensibility that is a part of the treatment - sensory re-education exercises - is possibly an important factor in such restoration of the fine-tuned receptive fields in the somatosensory cortex.

Our findings indicate that repeated cutaneous forearm anaesthesia over an eight-week period can improve hand function, focusing on sensation. With booster doses of EMLA^® ^once a month after the initial 8-week treatment we saw a long-lasting effect of up to 4 years in this group.

In a previous study on patients with median and ulnar nerve injuries EMLA^® ^treatment twice a week for two consecutive weeks resulted in a significant clinical improvement lasting at least four weeks after the EMLA^® ^application [25]. The mechanism behind this is probably a rapid unmasking of existing neural substrates but through a repeated deafferentation may create a more long lasting "time window" that will enable the patient to better consolidate the cortical changes thus producing a longer lasting improvement.

We would like to emphasise that the method described here, for improving sensory function in workers exposed to vibrating tools, in no way replaces ergonomic measures to minimize such exposure but may rather be seen as an additional measure.

Our observations open up interesting new perspectives regarding future possibilities for improving hand function and reducing disability in a group of patients, for whom no treatment is available today, using a simple and non-invasive method.

Our observations are encouraging, indicating a new principle for treatment of vibration-induced neuropathy of the hand, and perhaps also for other neuropathies. However, this is an initial series of cases in a long-term study, and the optimal protocol concerning time, dose, and practical handling of the cream aimed at achieving a long-lasting or permanent effect on sensory recovery has yet to be defined in larger randomized controlled studies.

## Competing interests

The authors declare that they have no competing interests.

## Authors' contributions

BR carried out the EMLA^®^) treatment. BR, AB and GL carried out the design of the study, analysis of data and drafting of this manuscript. All authors have read and approved the final manuscript.
